# Compete or Coexist? Why the Same Mechanisms of Symmetry Breaking Can Yield Distinct Outcomes

**DOI:** 10.3390/cells9092011

**Published:** 2020-09-01

**Authors:** Andrew B. Goryachev, Marcin Leda

**Affiliations:** SynthSys, Centre for Synthetic and Systems Biology, Institute for Cell Biology, University of Edinburgh, Edinburg EH9 3BD, UK; mleda@exseed.ed.ac.uk

**Keywords:** symmetry breaking, activator-substrate mechanism, mass-conserved models, cell polarity, pattern formation, small GTPases

## Abstract

Cellular morphogenesis is governed by the prepattern based on the symmetry-breaking emergence of dense protein clusters. Thus, a cluster of active GTPase Cdc42 marks the site of nascent bud in the baker’s yeast. An important biological question is which mechanisms control the number of pattern maxima (spots) and, thus, the number of nascent cellular structures. Distinct flavors of theoretical models seem to suggest different predictions. While the classical Turing scenario leads to an array of stably coexisting multiple structures, mass-conserved models predict formation of a single spot that emerges via the greedy competition between the pattern maxima for the common molecular resources. Both the outcome and the kinetics of this competition are of significant biological importance but remained poorly explored. Recent theoretical analyses largely addressed these questions, but their results have not yet been fully appreciated by the broad biological community. Keeping mathematical apparatus and jargon to the minimum, we review the main conclusions of these analyses with their biological implications in mind. Focusing on the specific example of pattern formation by small GTPases, we speculate on the features of the patterning mechanisms that bypass competition and favor formation of multiple coexisting structures and contrast them with those of the mechanisms that harness competition to form unique cellular structures.

## 1. Introduction

Cellular morphogenesis proceeds through a sequence of symmetry-breaking events resulting in the progressive increase in the complexity of cellular organization and function. For example, a mature neuron with its complex asymmetric arbor made of one long axon and multiple branched dendrites develops from a round precursor cell via such a sequence. Like the construction of a large and complex building begins with laying out of its foundation, cellular morphogenesis starts with a prepattern. This prepattern is typically made of proteins that were first distributed spatially homogeneously in the cytoplasm or nucleus and are then laid out in the shape of a desired pattern on a suitably “solid” support, such as the cell membrane. To explain morphological prepatterning, a powerful theory of activator-inhibitor and activator-substrate systems had been developed by the efforts of several generations of researchers [1,2]. For details, the reader is referred to several excellent reviews and books written on this sprawling topic [3,4,5,6,7,8,9,10,11]. The most popular patterns generated by these systems emerge via the diffusion-driven (aka Turing) instability of the spatially homogeneous state. They typically consist of spots and stripes and are known simply as “Turing patterns”. These patterns have two common properties that had been thought of as limiting their applicability to cellular morphogenesis. Firstly, once the conditions for the Turing instability are met, the spots or stripes emerge simultaneously everywhere there they can exist, for example, on the entire cell surface. Secondly, they form with an intrinsically defined characteristic size and, consequently, their number is determined by how many of them can fill out the whole cell surface. Contrary to these predictions, biological cells are known to develop fewer structures than what would fill the entire available space. At times, a cell produces only a single structure, such as the axon of a neuron [12]. Furthermore, those structures that are generated in multiple copies, like microvilli and filopodia, often appear to emerge sequentially, being added one by one to the preexisting number [13,14].

One avenue to resolving this discrepancy between the theory and the reality has emerged with the coming of age of fluorescence microscopy and the technology of fluorescent proteins. These experimental developments permitted researchers to closely follow cellular morphogenesis as it happens in live cells. This research showed that the morphogenetic prepattern, such as the polarized state of a chemotactic cell, can develop surprisingly fast, within just a few minutes. On such a short time scale, production and degradation of proteins comprising the prepattern is negligible and, thus, their cellular copy number can be considered constant throughout the establishment of the prepattern. This conservation of the total number (or the total mass) of the patterning proteins dramatically changes the character of the ensuing protein pattern. In brief, as the spots of the Turing pattern begin to develop by accumulating the patterning protein, they rapidly deplete the cytoplasmic store of the protein, making continued unrestrained growth of all spots impossible [15]. Larger spots manage to grow further but only at the expense of the smaller clusters that release their proteins back to the cytoplasm and eventually completely dissolve. This process of spot competition may proceed all the way to a single large spot that accrues all available protein. This ultimate super spot has no resemblance to the initial Turing pattern and its size and concentration profile cannot be predicted from the linear stability analysis of the homogeneous steady state that was disrupted by the Turing instability. This difference in the behavior of open and mass-conserved systems had long been generally known to mathematicians and physicists [16] but the recognition of its biological significance has not begun until recently [17,18]. Biological systems appear to exhibit the full spectrum of behavior from a vigorous competition invariably ending in the establishment of a unique cellular structure, such as the axon, to the formation of large arrays of similar structures, like microvilli, where no apparent competition between the individual structures is observed. Which properties of the symmetry-breaking mechanisms that underlie the formation of these structures are responsible for this difference? An appealingly simple answer to this question would be to propose that mathematically distinct mechanisms are responsible for different outcomes. Indeed, some early theoretical efforts apparently promoted this explanation. Recent comprehensive analyses of minimal two-variable models have greatly improved our quantitative understanding of the patterns formed by the mass-conserved systems and the phenomenon of competition between them. The picture that emerged from these analyses is more complex and nuanced. In a nutshell, quantitative differences, such as reaction constants, protein quantities and their ratios, rather than qualitative differences between the reaction mechanisms determine whether the system chooses competition or coexistence [19]. Unfortunately, a steep learning curve associated with the technical complexity of this research has inevitably reduced the ability of the broad research community to appreciate these results. Here we leave out rigorous derivations and, instead, review the main conclusions of the recent theoretical analyses focusing on their biological implications.

## 2. Yeast Bud and the Emergence of the Competition Concept in the Yeast Cell Polarity

To put abstract theoretical results into a concrete biological context, it is useful to introduce a specific cellular system in which the concept of competition plays an important role. Formation of the bud by the baker’s yeast, *Saccharomyces cerevisiae*, is arguably the best studied example of cellular morphogenesis where the mechanism of prepattern formation is known in sufficient detail. Since several excellent reviews are available on this topic [20,21], only the aspects essential to this contribution will be briefly outlined below.

Already early genetic screens identified two proteins that appeared to be right at the apex of the molecular network responsible for the bud formation—a small Rho GTPase Cdc42 and its activator, a guanine nucleotide exchange factor (GEF), Cdc24 [22,23]. Currently, informed by the years of cell biology research, we are confident that the prepattern for the insipient bud is a round spot formed on the budding yeast plasma membrane by the active, GTP-bound, form of Cdc42. Importantly, the peak concentration of total Cdc42 within the cluster is substantially higher than its background level elsewhere on the plasma membrane [24,25,26]. Thus, within this spot, Cdc42 is not only activated but is also highly concentrated (enriched).

From the theoretical point of view, Cdc42 and small GTPases in general are perfect molecular tools for the membrane-localized prepatterning of cellular morphogenesis [27,28,29]. Firstly, their ability to hydrolyze GTP and convert the released energy into a change in the protein conformation satisfies the essential prerequisite postulated already at the dawn of nonequilibrium thermodynamics [30,31]. Indeed, the reduction in entropy, which is associated with the formation of a pattern (i.e., symmetry breaking), needs to be coupled to the consumption of energy. Secondly, molecular properties of small GTPases allow them to shuttle between the cytoplasm and the membrane surfaces. Importantly, this shuttling is coupled to the activation-inactivation cycle (nucleotide cycling) so that, in general, the inactive form is cytoplasmic, while the active GTP-bound form is membranous [32]. This makes inactive GTPase an ideal embodiment of the “substrate” in the activator-substrate mechanism. All that remains to be done to crown active GTPase as the prototypical “activator” is to enable it with the property of autocatalytic activation. Many small GTPases, including Cdc42, indeed possess this crucial ability: they recruit onto the membrane their own activators, GEFs, thus enabling a positive feedback loop of autoactivation (for a recent review see [33]).

Specific to the field of bud morphogenesis, a dramatic breakthrough in the understanding of Cdc42 pattern formation was made by the discovery that Cdc42-GTP can recruit into the Cdc42 cluster its GEF Cdc24 with the help of Cdc42 effectors, molecules that specifically recognize and bind to the active GTPase [34,35,36,37]. The first mechanistic model of self-organized Cdc42 pattern formation [18] demonstrated that the experimentally characterized molecular interactions are both necessary and sufficient for the formation of the Cdc42 cluster via the Turing instability. It also demonstrated that the coupling of the activation-inactivation cycle of Cdc42 to its membrane-cytoplasmic shuttling leads to the substantial concentration of Cdc42 and its activator Cdc24 within the spot. As the total cellular number (mass) of these molecules is conserved, this accumulation leads to the depletion of their cytoplasmic pool—an important prerequisite for the competition. Finally, a biochemically detailed eight-variable model was systematically reduced to the two-variable model of the activator-substrate type. This model was distinct from the recently analyzed two-variable models in one important aspect. It conserved not only the total quantity of Cdc42 but also that of its GEF Cdc24. This cannot be achieved in a model that, from the beginning, has only two variables, the activator (Cdc42-GTP) and the substrate (Cdc42-GDP). However, since the two-variable model of [18] was derived from a mechanistically complete eight-variable model, conservation of GEF Cdc24 appeared in it in the form of a global negative feedback represented by an integral term that, incidentally, complicated the analysis of the model. As we will see in Section 4, this property largely determined the type of patterns produced by the model and their competition.

Analysis of the formation of a single Cdc42 spot in the model [18] showed that it emerges only as a final outcome of the temporary evolution of a fast succession of states with multiple small spots whose number rapidly decreased as their sizes increased. This coarsening behavior was explained by the vigorous competition of spots for the common cytoplasmic molecular pool (Cdc42 itself, but more importantly, its GEF Cdc24, and its effector Bem1) [18]. Furthermore, it was proposed that this competition, in fact, explains the normal uniqueness of the yeast bud. The fact that such a uniqueness (frequently named “singularity” in the literature) is necessary was well known, since the genetic material of the cell can be robustly divided between only two cells. However, the molecular mechanism of the uniqueness remained unknown. Interestingly, a few known genetic mutants breaking the rule of uniqueness pointed to the role of Cdc42 [38]. The modeling prediction of competition as the cause of the bud uniqueness was unexpected as no intermediate states with multiple Cdc42 spots had been observed before. Nonetheless, a dedicated effort to detect them by fast live-cell fluorescence imaging was successful, albeit it showed only a fleeting coexistence of two spots under normal (wild-type) conditions [39]. However, two different types of genetic perturbation aimed at weakening the spot competition produced the desired result—the simultaneous formation of two or more buds. Since these pioneering analyses, our understanding of competition between the yeast Cdc42 clusters has been substantially advanced both experimentally and theoretically, nevertheless, the problem continues to attract the interest of researchers [19,40,41,42,43].

## 3. Lessons from the Mass-Conserved Two-Variable Models

In this section we will survey the results of the theoretical analyses of the simplified two-variable activator-substrate models focusing on the most recent developments in this field. Keeping mathematical apparatus and jargon to the minimum, we will generally follow the Brauns et al. [44,45] approach that arguably provides the most comprehensive and self-consistent treatment of pattern formation in the mass-conserved activator-substrate (MCAS) models as seen from the physicist’s point of view.

A typical two-variable MCAS model consists of two partial differential equations:

(1) $$\left\{\begin{array}{c} \dot{u}=D_{u}\Delta u+F(u,v)\\ \dot{v}=D_{v}\Delta v-F(u,v) \end{array}\right.,$$

where u and v are the spatially and time dependent concentrations of the activator and the substrate, $\dot{u}$ and $\dot{v}$ their rates of change, $D_{u}\Delta u$ and $D_{v}\Delta v$ are the terms describing their diffusion, and F(u,v) is the reaction function. The fact that F(u,v) enters both equations but with opposite signs represents the mass-conserved nature of the model: any reaction that increases u, reduces v by the same amount, and vice versa. Typically, the reaction function can be written as:

(2) F(u,v)=A(u)v−I(u),

where A(u) and I(u) are two positive functions of the activator concentration that, keeping in mind our specific example of the small GTPase patterning system, can be called activation and inactivation functions, respectively [19,46]. Note that, because the MCAS model (1) is defined in a single spatial compartment (usually a 1D or 2D spatial domain that represents both the membrane surface and the mathematically “projected” on it cytoplasm), it cannot explicitly distinguish the membrane-cytoplasmic shuttling of GTPases from their activation-inactivation cycle. Instead, model (1) tacitly assumes that activation of a GTPase molecule attaches it to the membrane while inactivation instantaneously puts it back into the fast diffusing cytoplasmic pool.

In a spatially homogeneous steady state (SHSS) of model (1) the concentrations of u and v are constant in space and time and, thus, F(u,v) must be identically 0. Each SHSS is represented by just a single point on the phase plane (u,v) and we can find the positions of all SHSSs by solving equation F(u,v)=0. From (2) we readily get that all such states lie on the curve

(3) v=f(u)=I(u)/A(u).

The properties of this function that is but an explicit form of the reaction function F(u,v) largely determine the behavior of the model and, thus, f(u) deserves a closer look.

**Figure 1:**
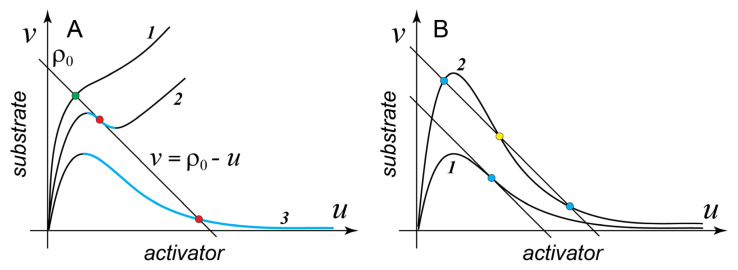
Properties of the reaction function F(u,v) on the phase plane (u,v). (**A**) Shape of the reaction function determines the behavior of the model (see text). Intersections of the reaction function with the mass conservation line $v=\rho_{0}-u$ define phase space locations of the model’s spatially homogeneous steady states. Green circle, SHSS (spatially homogeneous steady state) stable to the Turing instability. Red circles mark SHSS that lie on the descending fragment of the reaction function (cyan) and are unstable to the Turing instability. (**B**) Any MCAS (mass-conserved activator-substrate) model (1) whose reaction function has a descending fragment has a domain of parameters in which it is monostable in the well-mixed regime (case 1) and also a domain of parameters in which it is bistable (case 2). Stable states are shown by blue circles, unstable is shown by yellow circle.

Figure 1A shows several characteristic examples of f(u) with different shapes. Surprisingly, a simple mathematical analysis [45] provides an important result: 

**Result 1**. Only those MCAS models whose reaction function v=f(u) has a segment with negative slope on the (u,v) phase plane (descending segment, shown in color in Figure 1A) have the ability to form spatial patterns.

Thus, all models with a monotonously increasing f(u), such as case 1 in Figure 1A, cannot form a spatial pattern, while cases 2 and 3 can. More rigorously, for the spontaneous symmetry breaking to occur via the Turing instability, the negative slope of the descending segment should exceed $-D_{u}/D_{v}$. However, in most practical cases, the difference between the diffusion coefficients of the activator (a membrane protein) and that of the substrate (a cytoplasmic protein) is so large that this requirement does not change Result 1 appreciably, for example, for a molecule of small GTPase, typical diffusion coefficients are $\;D_{u}\approx 0.1\mu m^{2}/s$ and $D_{v}\approx 10\;\mu m^{2}/s$, so that $D_{u}/D_{v}=10^{-2}\approx 0.$ We now introduce two specific examples of the two-variable MCAS models that are frequently discussed and contrasted in the literature. Model A is a highly simplified version of the reduced two-variable model of the Cdc42 cluster formation introduced in [18]:

(4) $$Model\;\;A\;\left|\;F_{A}=(1+au^{2})v-bu;\;\;f_{A}(u)=\frac{bu}{1+au^{2}}\right.\;\;,$$

where a and b are the effective strengths of the positive feedback activation and inactivation, respectively. The activation part of its reaction function consists of two parts with distinct meaning. First term, a nonspecific constitutive background term (in this case, simply, 1), does not depend on the concentration of the activator and may represent a sum of spontaneous activation and activation by GEFs not involved in the positive feedback. Indeed, spontaneous, i.e., unassisted by enzymes, activation and inactivation are not negligible contributions to the nucleotide cycle of a small GTPase [47]. Second term, the classical autocatalytic function $au^{2}v$ [48,49] formally represents a nominal trimolecular reaction between the activator and the substrate:

(5) 2U+V→3U,

but in reality it stands for the autocatalytic activation that involves additional molecules, for example, the positive-feedback GEF (see [18] for an example of rigorous reduction of a detailed biochemical model with many variables). It is easy to see that $f_{A}(u)$ is Λ-shaped and behaves as case 3 in Figure 1A.

Model B is a dimensionless version [45] of the model first introduced in [50]:

(6) $$Model\;\;B\;\left|\;F_{B}(u,v)=\left(k+\frac{u^{2}}{1+u^{2}}\right)v-u;\;\;f_{B}(u)=\frac{u(1+u^{2})}{k+(1+k)u^{2}}\right.\;\;\;\;.$$

Here, a single nondimensional parameter k formally represents a constitutive substrate activation, which is independent of the activator. Comparing models A and B, we see that the sole difference between them is that the activation function in the model B is explicitly saturated and thus, at high concentrations of the activator, the rate of substrate activation is no longer dependent on the activator. As a result, $f_{B}(u)$ is generally N-shaped (case 2 in Figure 1A). However, $f_{B}(u)$ has a descending segment only for k<1/8. For larger k, this segment vanishes altogether and $f_{B}(u)$ acquires the shape shown as case 1, in other words, at k>1/8 model B can no longer form the pattern.

Prior to analyzing spatial patterns formed by the MCAS models, it is useful to have a closer look at the behavior of model (1) in the so-called “well-mixed regime”. Indeed, imagine that all biochemical reactions are happening in a shaken test tube. In this regime, spatial gradients are no longer possible, diffusion terms in the system (1) vanish, and the concentrations of the activator u(t) and inhibitor v(t) are only functions of time but not space. It is easy to see that the dynamics of the model in this regime is fully determined by the reaction function F(u,v) and the mass conservation relation:

(7) $$u(t)+v(t)=\rho_{0}.$$

On the phase plane (u,v), the mass conservation relation (7) is represented by a straight line with the slope −1 and the intercept $\rho_{0}$, a constant determined entirely by the initial concentrations u(0) and v(0) (see Figure 1A). Because of the mass conservation, in a well-mixed system the concentrations of the activator and inhibitor must remain on line (7) at all times. Therefore, all possible steady states of the well-mixed system lie on the intersection of the function f(u) and the mass conservation line (7). Importantly, the steady states of the model (1) in the well-mixed regime and SHSSs of the non-mixed system (1) are the same points on the phase plane (u,v). We now can extend Result 1 by stating that only those SHSSs of system (1) that lie on the descending segment of f(u) (but see the caveat following Result 1) can be destabilized by the diffusion-driven (Turing) instability and will *spontaneously* give rise to a spatial pattern (Figure 1A).

How many steady states can the MCAS model (1) have in the well-mixed regime? Figure 1A shows that f(u) always makes at least one intersection with the line of mass conservation. This, in fact, is required if model (1) is to describe a valid biochemical system. However, more intersections than one are possible. Consider model A at varying values of the inactivation rate b. Case 1 in Figure 1B shows that at sufficiently small b values there is only one intersection between the unstable (descending) segment of $f_{A}(u)$ and the mass conservation line. However, as b increases, the negative slope of $f_{A}(u)$ at its inflection point also increases [45] and at b>8 becomes more negative than the slope of the mass conservation line. For all larger b, model A is bistable: $f_{A}(u)$ intersects with the mass conservation line three times giving rise to two stable states and one (intervening) unstable state (Figure 1B). Analogously, in model B the function $f_{B}(u)$ intersects the mass-conservation line thrice for all k values smaller than ~0.0607 (precisely $3/2\sqrt{2}-1$). Thus, in the interval 0.0607<k<1/8 model B can form patterns but is not bistable. Therefore, both models have parameter domains where they can form spatial patterns but are not bistable in the well-mixed regime. The following important result [45,48] generalizes these examples:

**Result 2**. Bistability of the MCAS model (1) in the well-mixed regime is not necessary for the formation of spatial patterns at the same parameter values in the model without mixing. However, any MCAS model that can form patterns (has a descending segment of f(u), Result 1) is also bistable in the well-mixed regime within a parameter domain that overlaps but does not coincide with that of pattern formation.

This result finally settles the confusion that emerged in the early analyses of the two-variable MCAS model where it had been claimed that bistability of the reaction dynamics is required for the formation of patterns [50,51].

Now we discuss properties of the steady state patterns formed by the MCAS model (1) on extended spatial domains (of course, in the absence of mixing). If we add the two equations of model (1) together, we get that

(8) $$\dot{u}+\dot{v}=D_{u}\Delta u+D_{v}\Delta v.$$

For any steady state pattern (including SHSSs), both $\dot{u}$ and $\dot{v}$ are 0 by definition and we find that any steady state pattern must satisfy the equation

(9) $$D_{u}\Delta u+D_{v}\Delta v=0.$$

**Figure 2:**
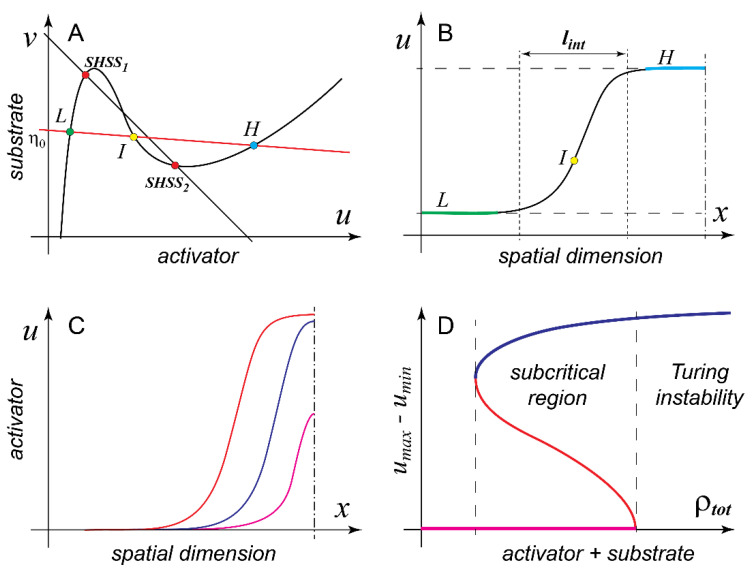
Spatial patterns formed by model (1). (**A**) Pattenrs formed by model B on a large spatial domain are determined in the phase plane (u,v) by the intersections of the reaction function and the line of patterns $v=\eta_{0}-D_{u}/D_{v}v$ shown in red (see text). Circles *L* and *H* represent low and high plateaus of the spatial pattern, circle *I* corresponds to the inflection point of the mesa pattern. Red circles represent steady states of the model in the well-mixed regime. (**B**) Mesa pattern formed by model B consist of low and high plateaus connected by interfaces with characteristic width $l_{int}$. (**C**) Model (1) forms stable peaks rather than mesas then it cannot reach saturation. Red curve represents mesa; blue curve represents a peak very close to saturation; magenta curve represents a peak far from saturation. Dash-dotted line in (**B,C**) represents an axis of pattern mirror symmetry. (**D**) Model (1) typically possesses a domain of parameters where both the SHSS (magenta) and the pattern (blue) are stable. Red curve represents unstable pattern (see text).

By a straightforward mathematical manipulation, (9) can be converted into

(10) $$v=\eta_{0}-\frac{D_{u}}{D_{v}}u,$$

an equation of another straight line on the phase plane (u,v) with a small negative slope $\left(-D_{u}/D_{v}\approx 0\right)$ and positive intercept $\eta_{0}$. Surprisingly, the fact that (9) holds true for any steady state pattern of MCAS (1) means that any such pattern, once projected onto the phase plane (u,v), must reside on line (10), which we can therefore call the “line of patterns”. To appreciate the importance of this line, let us consider an example. Figure 2A shows model B in the parameter domain (k=0.04) where it can form pattern and is also bistable in the well-mixed regime. Given that $f_{B}(u)$ is N-shaped, the line of patterns intersects $f_{B}(u)$ in three points. Since the reaction function F(u,v) becomes 0 everywhere on $f_{B}(u)$ and we consider a steady state (pattern), both the reaction and diffusion terms of model (1) must be identically 0 at the intersections of f(u) and the line of patterns (10). The two conditions can be satisfied together only if these points in the phase space (*L* and *H* on Figure 2A) correspond on the spatial domain to two flat plateaus with low and high concentrations of the activator and (nearly) the same concentration of the substrate (Figure 2B). The two plateaus are separated by an S-shaped interface and the third (intervening) intersection point of $f_{B}(u)$ and the line of patterns (point *I* on Figure 2A) corresponds precisely to the inflection point of this interface. Together with its mirror-symmetric counterpart, the interface shown in Figure 2B defines a pattern with a flat-topped profile, which is frequently referred to in the literature as mesa (Spanish for “table”). Note that the low and high plateaus of the mesa pattern are not identical to the two stable states of the well-mixed MCAS model (compare points L and H with SHSS1 and SHSS2 on Figure 2A). They coincide only if $D_{u}$ is equal to $D_{v}$, in which case the line of mass conservation and the line of patterns are the same line.

We now arrive at next important milestone [45] in the analysis of model (1)

**Result 3**. Mesa patterns are generic for the two-variable MCAS model (1). The only characteristic spatial size of the mesa pattern is the width $l_{int}$ of the interface between the low and the high plateaus of the mesa pattern and it is determined by the model’s intensive parameters, i.e., reaction constants and diffusion coefficients.

Indeed, the length (or the area in 2D) of the high plateau depends on the total quantity of the pattern forming protein in the system, in other words, an extensive parameter of the model. What does it mean, in practical terms, that these patterns are *generic* for the model? Firstly, we must assume that the linear size of the spatial domain L (e.g., the equatorial circumference of a spherical cell) is much larger than $l_{int}$ to permit formation of full-fledged mesas. Secondly, we stipulate that the total quantity of the patterning protein within the system should be sufficient to build at least one “minimal” mesa. Such a pattern would consist of two interfaces mirror-juxtaposed to each other with essentially no high plateau in between. Interestingly, several biologically important cases lie well outside of the parameter domain in which the requirements for the generic behavior are satisfied. Indeed, what if $l_{int}$ is on the same order as L and the total amount of the patterning protein in the cell is not sufficient to build even a minimal mesa pattern?

At this point in the discussion it is customary to switch from model B to model A. In model A, $f_{A}(u)$ and the line of patterns also have three points of intersection (unless $D_{u}$ is identically 0, or $D_{v}$ is infinite), but since $f_{A}(u)$ is Λ-shaped, rather than N-shaped, the third point of intersection that corresponds to the high plateau may lie at the unrealistically large value of the activator concentration. If we also assume that $l_{int}$ is smaller than L but is of the same order, a peak pattern whose maximum never reaches saturation (i.e., the value of the high plateau) can stably form in the system with mass conservation. If the pattern forming protein is continuously added to the system, the peak will also continue to grow both in height and in width until it finally reaches the saturation and becomes a mesa (Figure 2C). Unfortunately, peaks are not mathematically universal in the same sense as the interfaces of the mesa pattern and their analytical analysis is limited [45]. 

Can MCAS model (1) have a stable spatial pattern outside of the parameter domain of the Turing instability? In the above discussion, we concluded (Result 1) that only those SHSSs of the model that lie on the descending branch of f(u) can spontaneously break symmetry due to the amplification of the diffusion-induced spatial fluctuations. However, if we drop the requirement for the spontaneous symmetry breaking, stable patterns can be readily induced by finite perturbations well outside of the domain of Turing instability. This ability is associated with the so-called phenomenon of subcriticality, which effectively stands for the bistability between spatial patterns and should not be confused with the bistability of the well-mixed system. Figure 2D shows a schematic diagram illustrating this concept (see also Figure 1E and the accompanying discussion in [46]). Here a suitable order parameter, for example, the difference between the maximal and the minimal concentrations of the activator within the stable pattern, is plotted against one of the model parameters, for example, the total quantity of the pattern-forming protein in the cell. Figure 2D shows that subcriticality of the pattern corresponds to the interval of parameter in which both the SHSS and the pattern are stable. As is the case of all bistable systems, two stable states (a pattern and an SHSS, in this case) have to always be separated by an unstable state, which is also a pattern in this situation and can be numerically calculated with some computational tricks [45]. To induce a stable pattern in the bistable regime, the perturbation must exceed the unstable pattern and cannot be “small” in either its amplitude or spatial extent. In fact, it becomes larger the further the parameter value is from the boundary of the Turing instability. On the left (opposite to the Turing) end of the bistability interval, the required “perturbation” is essentially equal to the pattern itself. Subcritical patterns induced in model (1) by finite perturbations are also peaks and mesas and generally cannot be distinguished from their counterparts induced by the Turing instability. Rigorous analysis [45,48,52] shows that

**Result 4**. Bistability of the spatially homogeneous steady state and pattern is generic for MCAS model (1). Furthermore, any two-variable MCAS model (1) in which pattern can be induced by a finite perturbation also must have a domain of parameters in which it forms pattern via the Turing instability.

This result implies that, regardless of the exact mathematical formulation of the model, for example, the shape of f(u), the parameter domain in which model (1) forms pattern via the Turing instability is surrounded by a broader area of parameters in which the pattern can be induced by finite perturbations. Due to the nature of subcriticality (see Figure 2D), it is always found “on the fringes” of the domain of Turing instability. Perhaps, even more important is the second part of Result 4: patterns induced by spontaneous symmetry breaking (Turing instability) and the patterns induced by finite perturbations must always coexist in any model that is capable of forming pattern (albeit at different parameter values). In the words of Frank Sinatra, “you can’t have one without the other”. This means, for example, that there is no such model (1) that could only form patterns in response to finite perturbations but has no Turing instability. This settles another confusion in the field that has emerged from the claims that the subcritical formation (i.e., by finite perturbation) of mesa patterns in model B, so-called “wave pinning”, is a scenario fundamentally distinct from the Turing instability [50,51]. We now know that model B generates mesa patterns generically, via both Turing instability and by finite perturbations. Furthermore, like model A, it can also generate peaks, but unlike model A, it does so apparently only in the subcritical regime [45].

Finally, we are armed with sufficient apparatus to discuss competition between multiple maxima (“spots”) of the pattern generated by model (1). The interest in understanding the final outcome of pattern formation in the two-variable MCAS models has been largely, if not solely, driven by biological applications. However, this mathematical problem turned out to be a hard nut to crack. From the analysis of a few specific models, it had been conjectured that the coexistence of multiple spots in model (1) is always unstable and only a single spot would eventually emerge as the final outcome of the pattern coarsening process [17,18,53]. However, whether this hypothesis holds true for all possible models of type (1), remained unknown. Furthermore, the speed with which multiple spots can resolve into one is also of great importance for the biological systems. The prepattern for cellular morphogenesis is typically short-lived and is rapidly “fixed” by the downstream processes. Thus, if there is a strong biological reason why only a single spot needs to be generated, the competition must be really fast. For example, the Cdc42 clusters of the budding yeast had been observed to coexist for just a couple of minutes [39,42], while the leading protrusions of chemotactic *Dictyostelium* ameba coexist for an even shorter time, less than one minute (P.J.M. van Haastert, personal communication). Thus, what determines the kinetics of pattern coarsening was another unanswered question of high biological importance.

A major advance in the understanding of competition in model (1) was provided by the pioneering effort of Chiou et al. [19]. Firstly, the conjecture of ultimate instability of multiple spots in model (1) was mathematically proven for any reaction function F(u,v) in the practically important limit of infinitely fast diffusion of the substrate ($D_{v}\to \infty$). Secondly, and perhaps more importantly, it was demonstrated that the kinetics of competition is fundamentally dependent on the type of pattern. While peaks can compete essentially as fast as they form, mesas, once formed, are very static and can coexist for very long times. Ironically, mesa patterns were originally proposed as the model for the highly dynamic and rapidly competing protrusions of migrating cells [50,51]. Of course, peaks close to saturation exhibit intermediate competition kinetics. In fact, multiple peaks close to saturation will slowly compete to form several mesas that “compete” so slowly that, for all practical purposes, can be considered stably coexisting. Complementing results of [19], another recent work [44] built scaling arguments to approach competition from the physicist’s point of view. Using the average separation Λ between the maxima of the pattern as an effective order parameter that increases as the number of spots decreases, they showed that, for peaks, it increases as a power of the time of competition $t_{comp}$

(11) $$\Lambda \sim t_{comp}^{\alpha }\;,\;\;\;\alpha <1,$$

but for mesas it grows only as a logarithm of this time. Unfortunately, the mathematically non-universal nature of peaks, as opposed to mesas, showed itself also in this analysis. It transpired that the exponent α of the power law (11) is dependent on the exact definition of the reaction function F(u,v). This suggests a tantalizing hypothesis that some reaction mechanisms can provide faster peak competition than others. These results of [19] and [44] can be consolidated as the following

**Result 5**. All patterns with multiple maxima (spots) that are formed by two-variable MCAS model (1) are unstable, i.e., after passing of infinite amount of time they are guaranteed to resolve into a single spot (peak or mesa). However, the kinetics of this resolution process is qualitatively different for peaks and mesas.

This theoretical result comes with an important caveat. The validity of the assumption of mass-conservation demands that the characteristic time of pattern formation from its inception (either spontaneous or induced by a finite perturbation) to the resolution of competition should be much shorter than the characteristic times of production and degradation of proteins that are used to form the pattern. Ironically, it is not uncommon for the mathematical models to consider competition on time scales by far exceeding the entire lifetime of a biological organism. This raises an important question: how does the validity of Result 5 change if the mass-conservation is not (strictly) observed? Several works began to address this question by introducing production and degradation of the pattern-forming protein as small perturbations to the reactive dynamics [44,54,55]. Brauns et al. [44] demonstrated that this dramatically affects the first part of Result 5. Indeed, as the contribution of production and degradation to the reaction dynamics gradually increases, a pattern with multiple mesas becomes stable (i.e., mesas can coexist indefinitely). At even higher rates of production and degradation, mesas begin to split, making more, not less, spots. Taken together, these results cannot help but proffer an epistemological conclusion that theoretical models give simple predictions only when they are themselves, well, simple.

## 4. Implications for Cellular Morphogenesis

What can a biologist, who studies cellular morphogenesis in one of model organisms, learn about the competition and coexistence of cellular structures from the above theoretical results? Firstly, we conclude that, as far as the two-variable mass-conserved activator-substrate models are concerned, we are presently confident that all of them will eventually end up in the state with a single maximum. However, the time it takes the model system to arrive at this state could be anywhere between seconds and millennia. In real-life biological systems, “competition” and “coexistence” are more binary outcomes. If the “spots” of the prepattern compete any slower than the downstream morphogenetic processes that fix them, they might as well be considered as coexisting. Thus, in the budding yeast, formation of the Cdc42-GTP cluster triggers a cascade of processes, such as secretion of membrane vesicles, formation of septin cytoskeleton, softening and deformation of the cell wall, that together sculpt the nascent bud. If the competition between multiple Cdc42 clusters is slower than these processes, the cell will inevitably form multiple buds [39,41]. Thus, only “very fast” competition is of biological significance when a unique structure, for example, axon, bud, or protruding pseudopod, must form to fulfill its biological function within the cell.

It has been rightfully noted that the coexistence of multiple maxima in a pattern could be a stable state and not just a state of the frozen in time competition, as is the case for all two-variable MCAS models [40]. Several avenues to achieving stable coexistence are possible. For example, the requirement for the strict mass conservation can be relaxed [44,55]. Alternatively, additional negative feedback variables can be introduced [40,55]. Negative feedback is known to endow biological systems with homeostatic properties required for the stabilization of multiple structures. Specific to the small GTPase cell-patterning systems, negative feedback has been shown to generate oscillations and waves as well as blinking, jumping and moving spots of GTPase activity [42,56,57,58,59,60,61,62]. However, these rich dynamics and numerous models describing it lie outside of the scope of the present contribution.

While the two-variable MCAS models might provide a somewhat oversimplified description of actual cell-patterning systems, they offer clear recipes for designing patterning mechanisms with specific purpose. If multiple coexisting structures are the target, the “activator” state of the pattern-forming protein P must have minimal, if any, mobility $D_{u}\approx 0$. This can be readily achieved by polymerization, phase separation or simply by binding to the actin cytoskeleton and will ensure a sharp interface of the prepattern $l_{int}\ll L$, where L is a characteristic linear dimension of the cell. Reaction rates of the autocatalytic assembly of the “activator” cluster should evolve so that its “saturated” concentration (the high plateau of a mesa) is only moderately higher than the ground state concentration (the low plateau). Together, the two requirements will make for low and narrow mesas, hence, resulting in only a small number of patterning proteins per spot. If, in addition, P is an abundant cellular protein, formation of new spots will barely affect the existing ones.

If, however, only a unique cellular structure is required, which is the case of the establishment of cellular polarity, all requirements must be reversed. The “activator” should freely diffuse on the membrane and have a capacity to accumulate to high concentrations before reaching saturation. If not P itself, some component of the activation complex (see below) must be a small copy number protein, so that its accumulation within the pattern is acutely sensed by its cytoplasmic pool. These requirements are, indeed, highly consistent with the known experimental facts. In retrospect, it is not surprising, that much is known about the prepattern for the unique cellular structures, because they are themselves large, conspicuous entities marked by the high concentration contrast of the localization of marker proteins, which is readily detectable by the fluorescence microscopy. On the contrary, little is known about the prepattern for small repetitive structures, such as podosomes or microvilli. It is possible that, at least in part, this is because the fine-scale variation of the spatial distribution of the respective pattern-forming proteins is hard to resolve under the microscope and distinguish from the molecular noise.

Additional conclusions can be made for the specific case of small GTPase patterning systems, for which our understanding of the reaction mechanisms is much better than for other patterning systems. While in model (1) the type of pattern depends only on the total conserved quantity of the patterning protein, in the real GTPase systems, this will also be controlled by the ratio of the quantities of GTPase and its positive-feedback GEF. In the analysis of the behavior of the multivariable model for the Cdc42 cluster formation, it has been observed that the component present in the least amount defines the competition behavior of the pattern [40]. This fully confirms the early results of [18] that, following the contemporary experimental data, assumed that Cdc42 is in large molar excess over its positive-feedback activator GEF Cdc24 (~300:1). Because of that choice of parameters, pattern formation in model [18] stopped upon the depletion of the cytoplasmic store of the GEF and well below the saturation limit for Cdc42 insuring that only highly competitive peaks can be formed by this model. Relaxing this condition by reversing the GTPase:GEF ratio produced multiple coexisting mesa patterns, despite the reaction kinetics remaining the same. These modeling results are in good agreement with the results of [63] that, for the first time in any known system, succeeded in the in vitro reconstitution of the GTPase pattern formation using only a minimal set of recombinant proteins and lipid-coated beads. This remarkable effort produced multiple mesa-type spots of Rab5-GTP that stably coexisted during the duration of experiment. Remarkably, to achieve these patterns, the authors used molar excesses of the positive-feedback GEF Rabex-5 over the GTPase ranging from 5:1 to 50:1. At the latter value, the high plateau of the pattern covered most of the bead surface in perfect agreement with the theoretical predictions.

As a final remark, we would like to point out that the results discussed above need not be restricted only to the GTPase patterning systems or to the surface of membranes. A remarkable symmetry-breaking phenomenon in cells is the formation of a daughter centriole on the side of the mother centriole [64,65,66]. In this case, the pattern-forming protein is the kinase PLK4 and the “activator” is its phosphorylated and kinase-active state. Recently a competition-based model has been proposed to explain why under normal conditions only a single centriole forms, while overexpression of PLK4 generates multiple centrioles [67]. Interestingly, although this model is not mass-conserved, and, in fact, the growing PLK4 mass is the bifurcation parameter, the principle of competition can still be applied as far as the characteristic time scales of protein production and competition remain well separated.
